# Phthalates Impair Germ Cell Development in the Human Fetal Testis *in Vitro* without Change in Testosterone Production

**DOI:** 10.1289/ehp.11146

**Published:** 2008-09-09

**Authors:** Romain Lambrot, Vincent Muczynski, Charlotte Lécureuil, Gaëlle Angenard, Hervé Coffigny, Catherine Pairault, Delphine Moison, René Frydman, René Habert, Virginie Rouiller-Fabre

**Affiliations:** 1 Laboratory of Differentiation and Radiobiology of the Gonads, Unit of Gametogenesis and Genotoxicity, Commissariat à l’Energie Atomique, Direction des Sciences du Vivant, Institute of Cellular and Molecular Radiation Biology, Stem Cells and Radiation Department, Fontenay aux Roses, France; 2 Université Paris Diderot-Paris, Fontenay aux Roses, France; 3 Unité 566, INSERM, Fontenay aux Roses, France; 4 Service de Gynécologie-Obstétrique, Université Paris Sud, Hôpital Antoine Béclère, Clamart, France; 5 Unité 782, INSERM, Clamart, France

**Keywords:** human fetus, phthalates, testis development

## Abstract

**Background:**

Several studies have described an increasing frequency of male reproductive disorders, which may have a common origin in fetal life and which are hypothesized to be caused by endocrine disruptors. Phthalate esters represent a class of environmental endocrine-active chemicals known to disrupt development of the male reproductive tract by decreasing testosterone production in the fetal rat.

**Objectives:**

Using the organ culture system we developed previously, we investigated the effects on the development of human fetal testis of one phthalate—mono-2-ethylhexyl phthalate (MEHP)—an industrial chemical found in many products, which has been incriminated as a disruptor of male reproductive function.

**Methods:**

Human fetal testes were recovered during the first trimester (7–12 weeks) of gestation, a critical period for testicular differentiation, and cultured for 3 days with or without MEHP in basal conditions or stimulated with luteinizing hormone (LH).

**Results:**

Whatever the dose, MEHP treatment had no effect on basal or LH-stimulated testosterone produced by the human fetal testis *in vitro*, although testosterone production can be modulated in our culture system. MEHP (10^−4^ M) did not affect proliferation or apoptosis of Sertoli cells, but it reduced the mRNA expression of anti-Müllerian hormone. MEHP (10^−4^ M) reduced the number of germ cells by increasing their apoptosis, measured by the detection of caspase-3–positive germ cells, without modification of their proliferation.

**Conclusions:**

This is the first experimental demonstration that phthalates alter the development of the germ cell lineage in humans. However, in contrast to results observed in the rat, phthalates did not affect steroidogenesis.

Fetal life is a critical step in the development of male reproductive functions. Indeed, the two major functions of the testis, gametogenesis and steroidogenesis, take place during this period. In humans, testis formation begins by the migration of primordial germ cells (PGCs) from extraembryonic areas to the genital ridge during the fifth week of gestation (Wartenberg 1989). Sertoli cells then differentiate in the gonadal ridge and surround the germ cells to form the seminiferous cords between the sixth and seventh weeks (Gondos 1980; Wartenberg 1989). At this time, the PGCs are called gonocytes. In parallel, Leydig cells differentiate from mesen-chymal cells in the interstitial compartment (Habert et al. 2001). These steroidogenic cells are morphologically discernible at 8 weeks of gestation (Huhtaniemi and Pelliniemi 1992), whereas in organ culture, testosterone secretion is detected from 6 weeks (Lambrot et al. 2006). The appropriate onset of gametogenesis and steroido genesis is fundamental for the function of reproduction in the adult. Indeed, the number of germ cells formed during fetal life is essential for adult fertility. In mutant, germ-cell–deficient (*gcd*^−/−^) mice characterized by a reduced number of PGCs, as in mice lacking the *POG* (proliferation of germ cells) gene, the number of fetal germ cells is reduced and adult fertility is altered (Lu and Bishop 2003). In the same way, androgens and insulin-like factor 3 (Insl3) produced by fetal Leydig cells control the masculinization of the reproductive tract and genitalia (Jost et al. 1973; Kubota et al. 2002).

Several studies have described an increasing frequency of male reproductive dis orders in humans, such as a low sperm count and a resulting decline in fertility, increased incidence of testicular cancer, cryptorchidism, and hypospadias (reviewed by Bay et al. 2006; Sharpe and Irvine 2004). It has been suggested that these alterations are symptoms of a single entity called testicular dysgenesis syndrome (TDS) (reviewed by Sharpe 2003; Skakkebaek and Jorgensen 2005). It is currently thought that TDS is probably caused by changes in the development of the fetal testis and may result from the effect of genetic and/or environmental factors. Thus, TDS could result from exposure to environmental chemicals, which have steadily increased in diversity and concentration in the environment and food (Delbes et al. 2006; Skakkebaek et al. 2001). Several environmental chemicals are classed among the so-called endocrine disruptors. Many of them act on reproductive functions because of their estrogenic and/or antiandrogenic properties. In the present study we focused on the effects of phthalates (phthalic acid esters), which are industrial chemicals commonly found in many consumer products regularly used by humans, such as soap, shampoo, cosmetics, and hairspray. They are also used in flexible plastics, such as food and beverage packaging, children’s toys, and biomedical equipment (e.g., blood transfusion bags). Di-2-ethylhexyl phthalate (DEHP) is one of the most abundant phthalates produced (Latini et al. 2006). Latini (2005) demonstrated that phthalates, when administered orally to humans and rodents, are rapidly hydrolyzed by esterases in the gut and other tissues to produce the corresponding active monoesters. For example, DEHP is metabolized to its monoester metabolite, mono-2-ethylhexyl phthalate (MEHP), which is a recognized active testicular toxicant (Fisher 2004). Phthalates are not covalently bound to plastic products and therefore may leak out to contaminate blood or food products and can be ingested. In an epidemiologic study, 75% of the 289 human subjects tested were positive for the presence of four different types of phthalates in their urine samples (Blount et al. 2000). In rodents, both *in vivo* and *in vitro* approaches have been used to determine the effects on testicular functions of exposure to phthalates (reviewed by Sharpe 2006). Several studies have shown that fetal exposure to di(*n*-butyl) phthalate (DBP) induced by gavage of pregnant rats induces TDS-like effects (Barlow and Foster 2003; Fisher et al. 2003; Mylchreest et al. 2000).

However, despite the growing body of literature on phthalate reproductive toxicity and data demonstrating extensive human exposure, very few studies have examined the effects of these chemicals on human reproductive development. Recently, an inverse correlation has been shown between the maternal urinary phthalate concentration at the end of pregnancy and the anogenital distance at birth (Swan et al. 2005). In the same way, a dose-dependent association between phthalates in breast milk and levels of reproductive hormones in boys at 3 months of age has also been reported (Main et al. 2006). These findings are particularly important because they are the unique epidemiologic studies exhibiting negative effects of phthalates at environmental concentrations. Until now, no experimental study has succeeded in demonstrating a deleterious effect of phthalates on human testis functions or development.

In this study, we focused on phthalate effects specifically on the testis. We used the organ culture system of human fetal testes that we developed previously (Lambrot et al. 2006), coupled with morphologic, functional, and molecular methods (Lambrot et al. 2006, 2007), to analyze the effects of MEHP on the development of testicular somatic and germ cells during the first trimester of pregnancy (7–12 weeks of gestation). This early developmental period of the testis has been shown to be a critical window for the determination of the reproductive tract (Welsh et al. 2008).

## Materials and Methods

### Collection of human fetal testis

Human fetal testes were obtained from pregnant women referred to the Department of Obstetrics and Gynecology at the Antoine Béclère Hospital for legally induced abortion in the first trimester of pregnancy, that is, from the seventh until the twelfth week of gestation, as previously described (Lambrot et al. 2006). None of the terminations was for reasons of fetal abnormality, and all fetuses appeared morphologically normal. The sex of the fetus was determined by the morphology of the gonads, and the fetal age was evaluated by measuring the length of limbs and feet (Evtouchenko et al. 1996). The fetuses were dissected under a binocular microscope; testes were removed aseptically and immediately explanted *in vitro*. We found testes within the abortive material in only 12% of cases. The Antoine Béclère Hospital Ethics Committee approved this study.

### Organ cultures

We cultured testes on Millicell-CM Biopore membranes (pore size, 0.4 μm; Millipore, Billerica, MA, USA) as previously described (Habert et al. 1991; Lambrot et al. 2006). We used phenol red–free Dulbecco’s modified Eagle’s medium/ Ham F12 (1:1) (Gibco, Grand Island, NY, USA) supplemented with 80 μg/mL gentamicin (Sigma, St. Louis, MO, USA) and devoid of hormones, growth factors, and serum. We obtained MEHP from TCI Europe (Antwerp, Belgium).

Each human testis was cut into small pieces, and all pieces from the same testis were placed on Millicell membranes floating on 320 μL culture medium in tissue culture dishes. Tissues were cultured for 4 days at 37°C in a humidified atmosphere containing 95% air/5% carbon dioxide, and the medium was changed every 24 hr. We measured the responses to MEHP (10^−6^, 10^−5^, and 10^−4^ M) by comparing one testis cultured in medium containing the tested factor with the other testis from the same fetus cultured in control medium. We added luteinizing hormone (LH; 100 ng/mL) from human pituitary (≥ 5,000 IU/mg; Sigma) or ketoconazole (KTZ; 4 μM; Sigma) every 24 hr to the culture medium. Bromodeoxyuridine (BrdU 30 μg/mL; Amersham Biosciences, Little Chalfont, UK) was added during the last 3 hr of culture for the measurement of proliferating index. At the end of the culture period, explants were frozen in RLT buffer (Qiagen, Valencia, CA, USA) at −20°C for RNA analyses, or dry frozen with liquid nitrogen for protein analyses. For cellular analyses, the explants were fixed for 2 hr in Bouin’s fluid, embedded in paraffin, and cut into 5-μm sections.

### Germ cell counting

We mounted serial sections on slides, removed the paraffin, and rehydrated the sections. We then carried out immunohistochemical assays for anti-Müllerian hormone (AMH) as previously described (Lambrot et al. 2006) using an anti-AMH polyclonal antibody (1:2,000; generously provided by N. Di Clemente, INSERM U782, Clamart, France). We visualized peroxidase activity using 3,3′-diaminobenzidine as substrate. Germ cells were identified as AMH-negative cells within the seminiferous cords, whereas Sertoli cells were AMH positive. Counting was performed as previously described and validated for rodents (Livera et al. 2006; Olaso et al. 1998) and humans (Lambrot et al. 2006, 2007). Briefly, we counted germ cells in 1 of 10 sections for the 7-week-old human fetuses and 1 of 20 sections for later stages, but never fewer than 10 sections equidistantly distributed along the pieces of testis. We performed all counts using Histolab analysis software (Microvision Instruments, Evry, France). We counted all germ cells on the section and multiplied the sum of the values obtained for the observed sections of one testis by 10 or 20, respectively, to obtain a crude count of germ cells per testis. We then applied the Abercrombie formula (Abercrombie 1946), which uses the average measured diameter of the germ cell nuclei and the thickness of sections to correct for any double counting due to single cells appearing in two successive sections. All counts were carried out in a blind fashion.

### Immunohistochemical staining for cleaved caspase-3

Because caspase-3 is involved in most of the apoptotic pathways (Omezzine et al. 2003), we used immunodetection of caspase-3 to quantify the rate of apoptosis as previously described (Delbes et al. 2004; Lambrot et al. 2006). We mounted six sections on a single slide and heated the slide for 30 min in a permeabilization solution (0.05 M Tris, pH 10.6). The procedure was then the same as for detection of AMH, except that the primary antibody was anti-cleaved caspase-3, (1:50; Cell Signaling Technology, Beverly, MA, USA). Stained and unstained germ and Sertoli cells were counted in all six sections. For all immunohistochemical staining, we used tissue sections prepared without the primary antibody as negative controls.

### Measurement of BrdU incorporation index

We labeled testes with BrdU (labeling reagent diluted 1:100 according to the instructions of the cell proliferation kit; Amersham Biosciences) during the last 3 hr of culture. BrdU incorporation into proliferating cells was detected by immunocytochemistry according to the manufacturer’s recommendations, as previously described (Lambrot et al. 2006; Livera et al. 2000). The BrdU incorporation index was obtained by a blind counting of stained and unstained germ or Sertoli cell nuclei in all sections.

### Testosterone radioimmunoassay

We measured the testosterone secreted into the medium in duplicate by radioimmunoassay as previously described (Habert et al. 1991). No extraction or chromatography was performed because 17β-hydroxy-5α-androstan-3-one (DHT), the only steroid that significantly cross-reacts with testosterone (64%), is secreted in minute amounts by the fetal testis (George et al. 1987).

### Reverse transcription and real-time polymerase chain reaction

We performed RNA expression in the fetal testis by reverse transcriptase (RT) with the High Capacity cDNA Reverse Transcription kit (Applied Biosystems, Courtabeuf, France), followed by real-time polymerase chain reaction (PCR) as previously described (Lambrot et al. 2007). Primers and probes used were designed by Applied Biosystems: β-Actin primer, GenBank accession no. NM_001101.2 [National Center for Biotechnology Information (NCBI) 2008a]; AMH, Probe accession no. Hs00174915_ m1 (NCBI 2008b); Insl3, Hs01394273_m1; P450c17 (cytochrome P450 c17a), Hs00164375_m1; P450scc (cytochrome P450 11A1), Hs00167984_m1; RPLPO (large ribosomal protein PO), NM_053275.3; StAR (steroidogenic acute regulatory protein), Hs00264912_m1; and Wt1 (Wilms tumor 1), Hs01103749_m1. Reactions were carried out and fluorescence was detected using an ABI Prism 7000 apparatus (Applied Biosystems). Each sample was run in triplicate, and negative controls were run for every primer/probe combination. The measured amount of each cDNA was normalized using β-actin and RPLPO or Wt1 for AMH.

### Protein extraction and Western blotting

One testis was lysed in 20 mM Tris (pH 7.5), 150 mM NaCl, 1 mM EDTA, 1 mM EGTA, 1% Triton X-100, 2.5 mM Na_4_O_7_P_2_, 1 mM β-glycerol phosphate, 1 mM phenylmethylsul-fonyl fluoride, 1 mM Na_3_VO_4_, and 1 μg/mL leupeptin. Protein in total cell lysates (5 μg) was resolved by sodium dodecyl sulfate poly-acrylamide gel electrophoresis, electrophoretically transferred to a polyvinylidene difluoride membrane (Amersham Biosciences), and probed with antibodies for AMH (same as for immunohistochemistry) and β-actin (Sigma). We used Cy5-coupled anti-rabbit and Cy3-coupled anti-mouse secondary antibodies (Amersham Biosciences), and the blot was revealed under fluorescence in a Typhoon 9400 scanner (Amersham Biosciences). We quantified the bands by the volumetric method with the software ImageQuant (Molecular Dynamics, Sunnyvale, CA, USA).

### Statistical analysis

All values are expressed as mean ± SEM. For all mRNA expression analysis and studies on proliferation or apoptosis, we evaluated the significance of the differences between mean values for the treated and untreated testes from the same fetus using Wilcoxon’s nonparametric paired test (for small samples). For total germ cell number counting, we used Student’s paired *t*-test because of the high variability in the number of germ cell between ages. Concerning testosterone secretion analysis, we used one-way analysis of variance (ANOVA) to assess the significance of the differences for secretion evolution between control and treated testes during the 3 days of culture.

## Results

### Effect of MEHP on Leydig cell function

**Figure 1:**
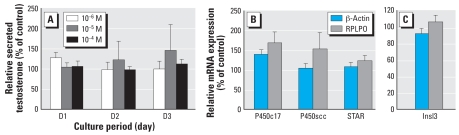
Effect of MEHP on Leydig cell function in the human fetal testis in culture. (*A*) Testosterone produced on days 1–3 (D1–D3) of culture measured by radioimmunoassay, compared with production on day 0. Values (mean ± SEM) are expressed as the percentage of the control value; MEHP 10^−6^ M, *n* = 4; MEHP 10^−5^ M, *n* = 3; MEHP 10^−4^ M, *n* = 15. (*B*) Levels of mRNA expression of specific Leydig cell markers using quantitative RT-PCR with specific primers to analyze expression of genes encoding P450c17, P450scc, StAR, and Insl3. Results (mean ± SEM of three different determinations) were standardized to either β-actin or RPLPO as the endogenous control and are presented as a percentage of the control value.

We cultured testes from fetuses at 7–12 weeks of development with or without 10^−6^, 10^−5^, or 10^−4^ M MEHP for 4 days. Daily testosterone production was unaffected by the addition of MEHP to the medium (Figure 1A). To check the lack of effect of MEHP, we analyzed the mRNA expression of various enzymes involved in steroidogenesis. MEHP treatment did not affect the mRNA expression of P450c17, P450scc, or StAR (Figure 1B). MEHP did not modify mRNA expression of Insl3 produced by fetal Leydig cells, which is known to be involved in testicular descent (Figure 1C).

**Figure 2:**
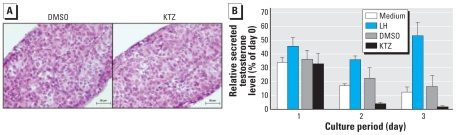
Effects of LH or KTZ on testosterone production by human fetal testis *in vitro*. (*A*) Photomicrographs of testes treated with 100 ng/mL LH or 4 μM KTZ show that KTZ was not toxic (bars = 30 μm). (*B*) Testosterone secreted in the medium at the end of days 1–3 of culture (mean ± SEM of three determinations) expressed as percentages of the secretion measured on day 0.

To assay the ability of testosterone secretion to be modulated in our organotypic culture system, we performed cultures with LH for testosterone stimulation and with KTZ for testosterone inhibition. With 100 ng/mL LH, the relative testosterone secretion was increased 5-fold at day 3 (Figure 2B). On the other hand, treatment with 4 μM KTZ (a cytochrome P450 inhibitor), which we have determined to be a nontoxic concentration for the testis (Figure 2A), induced very strong inhibition of testosterone production from day 2. Using one-way ANOVA, the change in testosterone secretion with both LH and KTZ treatments differed significantly from their respective controls. These results strengthen the validity of testosterone measurement in this model.

**Figure 3:**
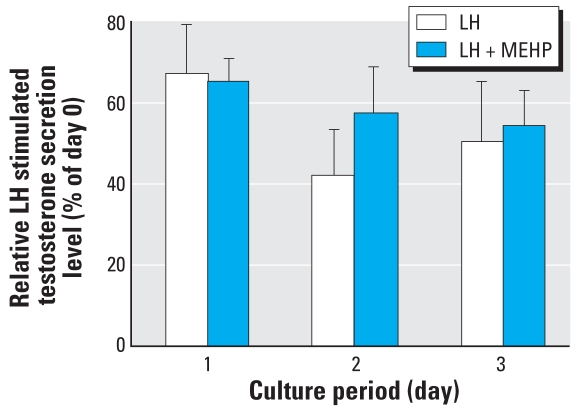
Effect of 10^−4^ M MEHP on LH-stimulated testosterone secretion by human fetal testes after 1–3 days of culture, expressed as percentage of the secretion measured on day 0 (mean ± SEM of seven determinations).

To investigate the effect of MEHP on stimulated testosterone secretion, testes were cultured in the presence of LH (100 ng/mL) with or without 10^−4^ M MEHP. Relative LH-stimulated testosterone production was unaffected by the addition of MEHP to the medium (Figure 3).

### Effect of MEHP on Sertoli cell development

**Figure 4:**
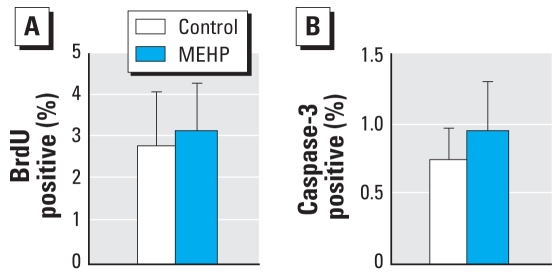
Effect of 10^−4^ M MEHP on the proliferative and apoptotic activities of Sertoli cells in cultured human fetal testes. (*A*) Percentage of Sertoli cells in proliferation, determined by incorporation of BrdU into the nuclei (*n* = 3). (*B*) Apoptosis of the Sertoli cells measured by immunodetection of the cleaved caspase-3 (*n* = 4). Values shown are mean ± SEM.

We studied the ratio of proliferative (BrdU positive; Figure 4A) and apoptotic (cleaved caspase-3 positive; Figure 4B) Sertoli cells after MEHP treatment for 3 days and observed that MEHP had no significant effect on these two activities.

**Figure 5:**
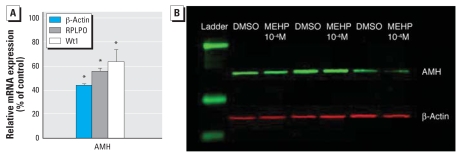
Effect of 10^−4^ M MEHP on AMH expression in cultured human fetal testes. (*A*) Results of quantitative RT-PCR with AMH-specific primers (mean ± SEM of three independent samples), normalized to β-actin, RPLPO, Wt1 (Sertoli endogenous control), expressed as a percentage of control. (*B*) Representative fluorescent Western blot revealing AMH protein (green) and β-actin (red). DMSO, dimethyl sulfoxide. *p < 0.05 in the paired comparison with the corresponding control values (Wilcoxon paired test).

We also analyzed the effect of MEHP on AMH expression by real-time RT-PCR (Figure 5A) and by fluorescent Western blotting (Figure 5B). Regardless of the housekeeping gene (β-actin or RPLPO) or specific Sertoli cell marker (Wt1, which is not significantly different in control and treated samples if standardized to β-actin), MEHP significantly decreased the mRNA level of AMH. However, the level of AMH protein standardized to β-actin was not modified by MEHP treatment.

### Effect of MEHP on fetal germ cell development

**Figure 6:**
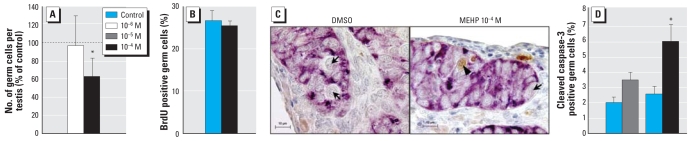
Effect of MEHP on the number and the proliferative and apoptotic activities of germ cells in cultured human fetal tesstes. (*A*) Total number of germ cells per testis expressed as a percentage of the value obtained with MEHP compared with the contralateral testis cultured in the absence of MEHP (mean ± SEM of four experiments). (*B*) Proliferation of the germ cells determined by immunohistochemical detection of BrdU incorporation into the nuclei during the last 3 hr of culture (mean ± SEM of three experiments). (*C*) Micrograph after immunohistochemical detection of cleaved caspase-3, a marker of apoptosis, after culture with or without MEHP. Arrowheads, cleaved caspase-3–positive gonocytes; arrows, cleaved caspase-3–negative gonocytes. Scale bars, 10 μm. (*D*) Percentage of labeled gonocytes for cleaved caspase-3 (mean ± SEM of three or four experiments). DMSO, dimethyl sulfoxide. *p < 0.05 in the paired comparison with the corresponding control values by Student’s *t*-test in (*A*) and Wilcoxon paired test in (*B*).

Addition of 10^−6^ M, 10^−5^ M, or 10^−4^ M MEHP for 3 days had no effect on the organization of the testis at the end of the culture (data not shown). Interestingly, regardless of the age of the fetus at explantation (from 7 to 12 gestational weeks), the higher dose of MEHP (10^−4^ M) significantly reduced the number of germ cells. Therefore, we expressed the results as a percentage of control and pooled the results from different ages (Figure 6A). However, the 10^−6^ M concentration had no effect.

Treatment with 10^−4^ M MEHP significantly increased the number of cleaved caspase-3–positive germ cells (Figure 6C,D) without altering their proliferation (Figure 6B). Treatment with 10^−5^ M MEHP increased, but not significantly, the number of cleaved caspase-3–positive germ cells (3.4% in the treated vs. 2% in the control testes) (Figure 6D).

## Discussion

In this study, we investigated the effect of one metabolite of phthalate ester, MEHP, on the development of human fetal testes, using our previously developed and validated organ culture system (Lambrot et al. 2006, 2007). In this organ culture system, the testicular architecture and intercellular communications are preserved enough to allow the development of the main fetal testicular cell types *in vitro*, without any added factor (Livera et al. 2006).

This approach allowed us to present here the first experimental demonstration that phthalates impair the development of the male fetal germ cell lineage in the human species. After 3 days of treatment, MEHP reduced by 40% the number of germ cells in cultured human fetal testis. This effect was due to a large increase in their apoptosis without modification of their proliferation. A negative effect of phthalates on gonocyte number has also been reported in rodents both *in vivo*, after gavage (Ferrara et al. 2006), and *in vitro*, in organ culture (Chauvigné F, Menuet A, Chagnon M-C, Lesné L, Jegou B, unpublished data; Lehraiki A, Szenker J, Habert R, Levacher C, unpublished data; Li and Kim 2003). It is interesting that, in rodents, the androgen pathway does not seem to be involved in germ cell number, because phthalates are distinct from flutamide in their ability to induce PGC degeneration (Mylchreest et al. 1999).

Phthalates induce the appearance of multi-nucleated gonocytes in rodents (Ferrara et al. 2006; Kleymenova et al. 2005). In the present study, we observed no multinucleated gonocytes in response to MEHP treatment. This may be due to a species characteristic; appearance of multinucleated gonocytes in rodents depends on the age of the fetus. For example, Ferrara et al. (2006) observed multinucleated gonocytes after DBP gavage only from day 19.5 postconception. Thus, the sensitive window in humans may occur later than the period studied here.

Phthalates are known as Sertoli cell toxicants in rodents. Some studies have reported a decrease in Sertoli cell number or proliferation (Hutchison et al. 2008; Li and Kim 2003; Li et al. 2000), interference with cytoskeleton (Kleymenova et al. 2005), and decrease in the expression of Sertoli cell markers (AMH, GATA4, inhibin) and of follicle-stimulating hormone–stimulated cAMP production (Fisher et al. 2003; Heindel and Chapin 1989; Hutchison et al. 2008; Li and Kim 2003). In this study, we observed that MEHP did not affect the proliferation or apoptosis rate of Sertoli cells. On the other hand, MEHP decreased the mRNA expression of AMH whether we normalized relative expression to ubiquitous expression or to the Sertoli gene. Surprisingly, however, the intracellular AMH protein level was not modified by MEHP treatment when analyzed by Western blot. To explain this discrepancy, we can hypothesize that MEHP affects RNA expression earlier than it does protein expression.

In the rat, it is currently thought that phthalates act mainly on fetal Leydig cells. Thus, the main effects of phthalates are the suppression of testosterone production, an abnormal Leydig cell aggregation, and the presence of intratubular Leydig cells (Culty et al. 2008; Fisher et al. 2003; Parks et al. 2000). Jegou and colleagues (Chauvigné F, Menuet A, Chagnon M-C, Lesné L, Jegou B, unpublished data) have also observed a similar decrease in testosterone production in organ culture of rat fetal testis, and we confirmed this result (data not shown). Nevertheless, Hallmark et al. (2007) have shown that MBP (monobutyl phthalate) reduces human chorionic gonadotropin–stimulated but not basal steroidogenesis in rat fetal testis explants. In the present study, we observed that MEHP changes neither the basal nor the LH-stimulated production of testosterone by the human fetal testis in culture. This was confirmed by the absence of modification of the mRNA levels of steroidogenic enzymes after MEHP treatment. Hallmark et al. (2007) also found no *in vitro* effect of phthalates (MBP and DBP) on steroidogenesis of human fetal testis explants recovered during the second (15–20 weeks) trimester of pregnancy. So, steroidogenesis of the human fetus during the first and second trimesters seems not to be sensitive to phthalates. This period in humans corresponds to the window studied in the rat model. It is interesting that, in mice, MEHP stimulates testosterone production both in the fetal testis (Gaido et al. 2007; Lehraiki A, Szenker J, Habert R, Levacher C, unpublished data) and in Leydig tumor cells (Gunnarsson et al. 2008). This lack of effect on testosterone production in humans suggests differences between species.

The results presented here contrast with epidemiologic data in humans (Main et al. 2006; Swan et al. 2005). We can formulate two hypotheses to explain this apparent discrepancy: first, the studies differ in phthalate concentration, nature, and duration of exposure. We chose to investigate the effect of phthalate concentrations ranging from 10^−6^ M to 10^−4^ M. In a prospective study on cryptorchidism carried out on the phthalate monoester contamination of human breast milk, Main et al. (2006) found many phthalate monoesters over a large concentration range, from 1.5 to 1,410 μg/L, which corresponds to about 10^−9^ M to 10^−6^ M for MEHP. In our study, we observed no effect at the lower concentration, either on steroidogenesis or on gametogenesis. However, the mother and thus the fetus are exposed to a combination of multiple phthalates (Swan et al. 2005), which could explain the need for a greater dose of MEHP to show a potent effect *in vitro*. Second, in our study we focused on the effect of MEHP specifically on testis, so we cannot rule out that the observed effects in epidemiologic studies (cryptorchidism and anogenital distance) are due to a direct effect of phthalates on the reproductive tract.

In the human fetus, intraabdominal testicular descent to the inner inguinal ring is initiated at about 10–14 weeks of gestation (Barteczko and Jacob 2000; Klonisch et al. 2004). A role for Insl3 secreted by differentiated Leydig cells, in development of the gubernaculum and this first phase of testicular descent, has emerged after analysis of mice genetically modified for Insl3 expression (Adham et al. 2002; Ivell and Bathgate 2002; Nef and Parada 1999; Zimmermann et al. 1999). Moreover, underdeveloped gubernaculum (Barlow and Foster 2003) and reduced Insl3 expression have been observed after fetal exposure to several different phthalates in male rats (Lehmann et al. 2004; McKinnell et al. 2005; Wilson et al. 2004). We observed no effect of 10^−4^ M MEHP (highest concentration) on the Insl3 mRNA in human testis culture, even though our study took place during the setup of the testicular descent. Laguë and Tremblay (2008) have recently demonstrated that MEHP represses Insl3 transcription by antagonizing testosterone action in Leydig cells. Thus, the absence of effect on Insl3 expression observed here can be explained by the lack of effect of MEHP on testosterone production in our model. Nevertheless, androgen receptor antagonists seem to have no effect on Insl3 expression (McKinnell et al. 2005; Wilson et al. 2004).

In conclusion, this is the first experimental demonstration that phthalates, a family of compounds known as endocrine disruptors, widely distributed in the environment, are able to alter the development of male germ cell lineage in humans. This effect is not mediated by a decrease in the testosterone produced by the Leydig cells, which is unchanged. Furthermore, this study shows the efficiency of our organ culture system in investigating the effects and mechanisms of action of environmental disruptors on the development of the human fetal testis. Lastly, our work provides important insight into the potential role of exposure to environmental pollutants during fetal testicular development and their potential deleterious effects on male fertility in adulthood.
